# Genomic analysis identifies candidate supergene architecture for host-feeding behavior in wild *Aedes aegypti* dengue vector mosquitoes

**DOI:** 10.3389/fgene.2026.1910877

**Published:** 2026-09-09

**Authors:** Devojit Kumar Sarma, Sudeesh Warkare, Sweta Mishra, Deepanker Das, Lokendra Rathod, Manoj Kumawat, Siddharth Singh Tomar, Samradhi Singh, Manoj Kumar, Rajnarayan R. Tiwari

**Affiliations:** 1 ICMR-National Institute for Research in Environmental Health, Bhopal, India; 2 Indian Institute of Science Education and Research, Bhopal, India

**Keywords:** *Aedes aegypti*, anthropophilly, chromosomal inversion, genome wide association studies (GWAS), odorant receptor, single nucleotide polymorphisms (SNP)

## Abstract

**Background:**

*Aedes aegypti* is the primary arboviral vector globally, and its affinity to feed on human hosts is a key determinant of transmission intensity. Despite the epidemiological importance of anthropophily, the genomic architecture underlying the molecular basis of host-feeding behavior remains understudied.

**Methods:**

Here, we present an integrated genome-wide association study (GWAS) and structural variant analysis of host-feeding behavior in 21 field-collected *Ae. aegypti* females phenotyped as human-blood-fed (HF; n = 11) or non-human-blood-fed (NHF; n = 10). Whole-genome sequencing yielded 661,519 high-quality SNPs distributed across all three chromosomes. Principal component analysis on a linkage disequilibrium-pruned dataset revealed modest population structure (PC1 = 11.69%, PC2 = 10.98%).

**Results:**

GWAS using a general linear model with five PC covariates (λ = 1.05) identified 11 suggestive SNPs in genes including an actin binding protein on chromosome 2 and G-protein coupled receptor 39 on chromosome 3. Chromosomal inversion analysis using Delly identified an HF-specific inversion on chromosome 2 harboring 13 genes across three functionally coherent chemosensory categories: the sensory developmental regulator tap, odorant receptors *(Or6, Or31, Or33)* and an odorant-binding protein (*Gp68*), and eight gustatory receptor genes (*Gr15–Gr19a, Gr35, Gr36, Gr66*).

**Conclusion:**

The concentration of co-adapted chemosensory genes within a single non-recombining chromosomal unit is consistent with a possible supergene model of anthropophily, paralleling inversion-mediated behavioral divergence in *Anopheles gambiae*. These exploratory findings provide a preliminary genomic framework for host-seeking behavior in Ae. aegypti and identify probable candidate loci warranting functional validation before application to vector surveillance.

## 1 Introduction

*Aedes aegypti* is one of the most significant mosquito vectors globally, responsible for the transmission of several arboviruses of major public health concern, including dengue, chikungunya, Zika, and yellow fever. The global expansion of dengue, in particular, has reached alarming proportions; in 2024 alone, a historic high of over 14.6 million cases and more than 12,000 dengue-related deaths were reported, affecting over 100 countries on all continents (WHO, 2025). Updated global risk estimates suggest that the emergence and global expansion of *Ae. aegypti* now puts more than half of all humans at risk of arbovirus infection, a figure substantially higher than earlier projections (Bhatt et al., 2013) This epidemiological burden is tightly coupled to the ecology and behavior of *Ae. aegypti*, a species that has evolved a strong preference for human hosts, thrives in urban environments, and exhibits efficient virus transmission dynamics (Kraemer et al., 2015). Among the behavioral traits that underpin its vectorial capacity, host-seeking and blood-feeding preferences are particularly critical, as they directly determine human–vector contact rates and thus transmission intensity (Crawford et al., 2025).

The current body of knowledge shows that the host preference in mosquitoes is a complex, multifactorial phenotype shaped by environmental cues, sensory perception, and genetic architecture. In *Ae. aegypti*, anthropophily, which is the preferential feeding on humans, is a key evolutionary adaptation that enhances its efficiency as a disease vector, and is present to markedly different degrees across populations. Two recognized subspecies supports much of this variation; the cosmopolitan, highly anthropophilic *Ae. aegypti aegypti* and the ancestral, zoophilic *Ae. aegypti formosus*, which persists in sub-Saharan Africa. Recent work suggests that human specialization originally evolved approximately 5,000 years ago as an adaptation to the extremely long, hot dry seasons of the Sahel region of West Africa, where scarcity of natural aquatic habitat forced *Ae. aegypti* to rely on human-stored water for breeding, driving co-evolutionary adaptations to both artificial containers and human hosts (Rose et al., 2023) This specialization involves a sophisticated integration of olfactory, visual, and thermal cues, enabling mosquitoes to discriminate between human and non-human hosts (Montell, 2025). Previous work has identified specific olfactory receptors, most notably Or4, which confers enhanced sensitivity to sulcatone, a human-associated volatile, and odorant-binding proteins responsive to skin microbiota-derived compounds as key mediators of host discrimination (Degennaro et al., 2013; McBride et al., 2014). However, despite these mechanistic advances, the broader genetic architecture underlying natural variation in host preference remains poorly resolved (Rose et al., 2023).

Most of the existing knowledge on mosquito host-seeking behavior has been derived from laboratory-reared colonies, which are often subject to strong artificial selection, inbreeding, and environmental homogenization (Clark et al., 2011) While these systems provide controlled conditions for mechanistic studies, they fail to capture the genetic diversity and ecological complexity present in wild populations (Lainhart et al., 2015). Field populations of *Ae. aegypti* are shaped by heterogeneous environments, diverse host availability, and local adaptation pressures, all of which can influence feeding behavior. Recent population genomics work has shown that genome-wide ancestry is predictive of human specialist behavior in African populations, with a subset of the genome offering even greater predictability, underscoring the potential of field-based genomic approaches to dissect this trait (Main et al., 2016; Crawford et al., 2017). Consequently, there is a compelling need to investigate the genomic basis of host preference in natural populations to better understand the evolutionary and epidemiological dynamics of this phenotype.

Recent advances in high-throughput sequencing and population genomics have opened powerful new avenues for dissecting complex behavioral traits in non-model organisms. Genome-wide association studies (GWAS) offer a robust framework for identifying SNPs and genomic loci associated with phenotypic variation, including behavioral traits such as host preference. In *Ae. aegypti*, GWAS approaches have been successfully applied to insecticide resistance: using the *Ae. aegypti* 50k SNP chip (Cosme et al., 2022), identified new loci associated with pyrethroid resistance in Brazilian populations, demonstrating the feasibility of association mapping for quantitative traits in this species. Their application to host-seeking behavior, however, remains largely unexplored. In parallel, structural genomic variants such as chromosomal inversions are increasingly recognized as important drivers of adaptation, maintaining co-adapted gene complexes and facilitating local adaptation under gene flow (Kirkpatrick and Barton, 2006). Linked-read sequencing of *Ae. aegypti* has identified a library of 32 novel chromosomal inversions, with many microinversions found to have introgressed across subspecies and to have captured clusters of odorant-binding protein genes that may contribute to host feeding preference, pointing to structural variation as a potentially important but understudied component of the host preference genomic landscape (Redmond et al., 2020).

Understanding the genomic determinants of human blood-feeding behavior is particularly important in the context of vector control. Traditional strategies primarily target mosquito abundance through insecticides or source reduction; however, behavioral traits such as host preference can modulate transmission risk independently of population density. A genomic predictor of host preference could serve as a point of potential interventions for functional studies and provide predictive markers that reduce reliance on labor-intensive behavioral assays when estimating vectorial capacity; moreover, ecological models incorporating such genomic data have already shown the ability to predict arboviral seroprevalence variation at regional scales. Here, we investigate the genomic basis of host preference in field collected *Ae. aegypti* populations with the aim of identifying SNPs, candidate loci, and structural variants associated with anthropophily.

## 2 Methods

### 2.1 Mosquito sampling strategy

Suitable collection sites for *Ae. aegypti* were identified within Bhopal city, Madhya Pradesh, India, comprising mixed habitats where human habitations and cattle sheds coexisted in close proximity. This site selection aimed to ensure the simultaneous availability of multiple blood-meal sources within a shared microhabitat and minimize biases related to incomplete penetrance (Main et al., 2016). Because the relative abundance and exact spatial distribution of humans versus animals were not quantitatively measured, we note that the identified blood-meal sources represent realized feeding behavior rather than a strict measure of innate genetic preference. To further mitigate the effects of temporal environmental heterogeneity on host choice, sampling was restricted to a single season. The collected specimens were immediately stored at 4 °C, transported to the laboratory, and processed according to the protocol detailed by Sarma et al. (2022).

### 2.2 Genomic DNA library preparation and illumina sequencing

A total of 21 field-collected *Ae. aegypti* females confirmed as *Ae. aegypti* by morphological keys were processed for whole-genome sequencing. Following DNA extraction, quantity was assessed using a Qubit 4.0 fluorometer (Thermo Fisher Scientific, Massachusetts, USA) with the dsDNA High Sensitivity Assay Kit, and quality was evaluated by agarose gel electrophoresis. All 21 samples met minimum quality thresholds and were confirmed to be *Ae. aegypti* by species-diagnostic PCR. Blood meal host identity was determined by cytochrome *b* PCR amplification and Sanger sequencing, and individuals were categorized as human-blood-fed (HF; n = 11) or non-human-blood-fed (NHF; n = 10). Since this phenotypes reflect realized feeding outcomes shaped jointly by innate host-seeking bias, host availability microhabitat distribution, and opportunistic encounter, we refer to the blood-meal-derived classification as field host-feeding phenotype (human-fed, HF vs. non-human-fed, NHF).

Libraries were prepared using the Nextera DNA Flex Library Preparation Kit (Illumina, San Diego, CA, USA) with 25–50 ng of input DNA per sample, following the manufacturer’s protocol. Dual indexing was performed using TruSeq CD Index adapters (Illumina) to enable sample multiplexing. Fragment size distributions of the final libraries were assessed on a TapeStation 4150 system (Agilent Technologies) using High Sensitivity D1000 ScreenTapes (Agilent, #5067-5584), and final molar concentrations were determined using the Qubit dsDNA HS Assay Kit (Thermo Fisher Scientific, #Q32851). Individually barcoded libraries were sequenced on an Illumina NovaSeq 6,000 platform (PE150 mode), generating approximately 424 Gb total data, corresponding to approximately 15.77x mean genome coverage of the *Ae. aegypti* reference genome (∼1.28 Gb).

### 2.3 Read processing and variant calling

Quality assessment of raw reads was performed using FastQC v0.12. Adapter trimming and quality filtering were carried out with Trimmomatic v0.36 using the following parameters: ILLUMINACLIP:TruSeq3-PE.fa:2:30:10, LEADING:3, TRAILING:3, SLIDINGWINDOW:4:15, MINLEN:50; reads were discarded if they contained >10 ambiguous (N) bases or fell below a mean Phred quality score of 20. Cleaned reads were aligned to the *Ae. aegypti* reference genome assembly AaegL5 (GenBank accession: GCA_002204515.2; Matthews et al., 2018) using BWA-MEM v0.7.17 with default parameters. The AaegL5 assembly was selected as the current community-standard reference, offering substantially improved contiguity and chemosensory gene annotation relative to earlier assemblies. Repetitive regions of the reference were soft-masked prior to alignment to reduce spurious multi-mapping; SNPs located in masked regions were subsequently excluded from downstream analyses.

Aligned reads were coordinate-sorted using SAMtools v1.21, and PCR duplicates were marked and removed using Picard MarkDuplicates v3.0. Per-sample alignment statistics, including mean depth and mapping rate, were computed with SAMtools flagstat and Qualimap v2.2 to confirm adequate coverage across samples. Per-sample variant calling was performed using GATK HaplotypeCaller v4.6 in GVCF mode (--emit-ref-confidence GVCF), following GATK Best Practices for germline short variant discovery. Per-sample GVCFs were consolidated using GenomicsDBImport and jointly genotyped using GenotypeGVCFs, enabling multi-sample likelihood integration that improves sensitivity and specificity relative to single-sample calling. The resulting multi-sample VCF was subjected to hard filtering, retaining SNPs that met the following criteria: biallelic only (excluding multi-allelic sites); mean read depth ≥10; minor allele frequency (MAF) ≥ 0.05; Hardy–Weinberg equilibrium (HWE) p-value ≥10^–3^; and QUAL score ≥30. Filtering was implemented using VCFtools v0.1.16, BCFtools v1.13, and PLINK v1.9. InDels were retained separately for structural annotation but excluded from the GWAS analysis.

### 2.4 Population structure analysis

Prior to association testing, population structure among the 21 *Ae. aegypti* samples was assessed by Principal Component Analysis (PCA) to identify and account for cryptic stratification that could confound GWAS results. The filtered SNP dataset was first pruned for linkage disequilibrium (LD) using PLINK v1.9 (window size: 50 SNPs, step size: 10, r^2^ threshold: 0.2) to ensure that PCA eigenvectors reflect population ancestry rather than local LD structure. PCA was computed on the LD-pruned dataset using the--pca function in PLINK v1.9. To objectively justify the number of PCs incorporated as covariates in the GWAS model, a Scree plot of the eigenvalue distribution was generated to evaluate the variance explained by each component. To avoid over-parameterization and the severe reduction of residual degrees of freedom, a critical risk due to the small sample size (n = 21), the final number of PCs was determined empirically by balancing the variance explained with the resulting genomic inflation factor (λ). For visual representation of the primary axes of population structure, the first two principal components were extracted and visualized using ggplot2 in R v4.3.

### 2.5 Genome-wide association study

GWAS was performed using the R package rMVP (Yin et al., 2021) to identify SNPs associated with host-feeding phenotype (HF vs. NHF, coded as a binary trait). The General Linear Model (GLM), incorporating the first *k* PCs (as determined above) as fixed-effect covariates was used. The genomic inflation factor (λ) was computed from the GLM results to assess residual stratification and validate the covariate selection. Models demonstrating severe deflation or inflation due to sample-size constraints were scrutinized, and the optimized PC model was prioritized. Genome-wide significance was defined by a Bonferroni-corrected threshold of α = 0.05/N, where N is the total number of SNPs passing quality filters. Given the limited statistical power conferred by the sample size, a secondary suggestive threshold of p < $1.0\times 10^{-5}$ was also applied to identify candidate loci for functional follow-up (Hammond et al., 2021). Manhattan and quantile–quantile (Q-Q) plots were generated within rMVP to visualize association results and evaluate model calibration. Linkage disequilibrium (LD) decay around significant and suggestive SNPs was characterized using PLINK v1.9 (--r2 --ld-window-kb 500) to define haplotype block boundaries and identify candidate gene regions. Gene Ontology (GO) enrichment analysis of candidate genes within associated loci was performed using DAVID (https://david.ncifcrf.gov/) and STRING v12.0 (https://string-db.org/).

### 2.6 Detection of chromosomal inversions

Chromosomal inversions were identified from the deduplicated BAM files using Delly v2.0 (Rausch et al., 2012), which detects structural variants by integrating discordant paired-end read orientations and split-read alignments. All 21 samples were processed individually under default parameters, and per-sample VCF outputs were merged. Population-level genotyping was performed across all samples to distinguish inversions supported by multiple individuals from singleton or artefactual calls. Only inversion calls passing the “PASS” filter criterion, supported by a minimum of five supporting reads (paired-end and/or split-read evidence combined), and spanning at least 50 bp were retained for downstream analysis. Calls overlapping known repeat regions or segmental duplications in the AaegL5 assembly were excluded to reduce false positives arising from the highly repetitive *Ae. aegypti* genome. Inversion calls were partitioned by phenotypic group (HF vs. NHF) to identify structural variants with group-differential frequencies. Genes located within inversion boundaries exclusive to, or significantly enriched in, one phenotypic group were identified and functionally annotated using DAVID and STRING v12.0. Visual validation of inversion events was performed using the Integrative Genomics Viewer (IGV) v2.19, examining paired-end orientation patterns and split-read alignments at predicted breakpoints (Supplementary Figure S1).

### 2.7 Statistical analysis

Pairwise comparisons of quantitative metrics between the HF and NHF groups were assessed using Student’s t-test, with statistical significance defined at p < 0.05. Where comparisons involved more than two groups or multiple metrics, one-way ANOVA was applied followed by appropriate post-hoc testing. All statistical analyses were performed in Minitab v21.

## 3 Results

### 3.1 Genome sequencing and read mapping

A total of 21 *Ae. aegypti* females, 11 confirmed human-blood-fed (HF) and 10 non-human-blood-fed (NHF), passed all quality control criteria and were included in whole-genome sequencing. Sequencing on the Illumina NovaSeq 6000 platform generated a total of approximately 212 Gb of high-quality paired-end reads across all 21 samples. The mean read length after quality trimming was 147 bp, and the GC content of the trimmed reads was 38.5%, consistent with the published *Ae. aegypti* genome composition (∼38%); (Matthews et al., 2018). A mean of 98.9% of reads per sample were successfully aligned to the AaegL5 reference genome (GCA_002204515.2), indicating high mapping efficiency and confirming the species identity of the samples. The mean genome-wide sequencing depth was 15.77 x per sample.

### 3.2 Detection and distribution of SNPs

**FIGURE 1:**
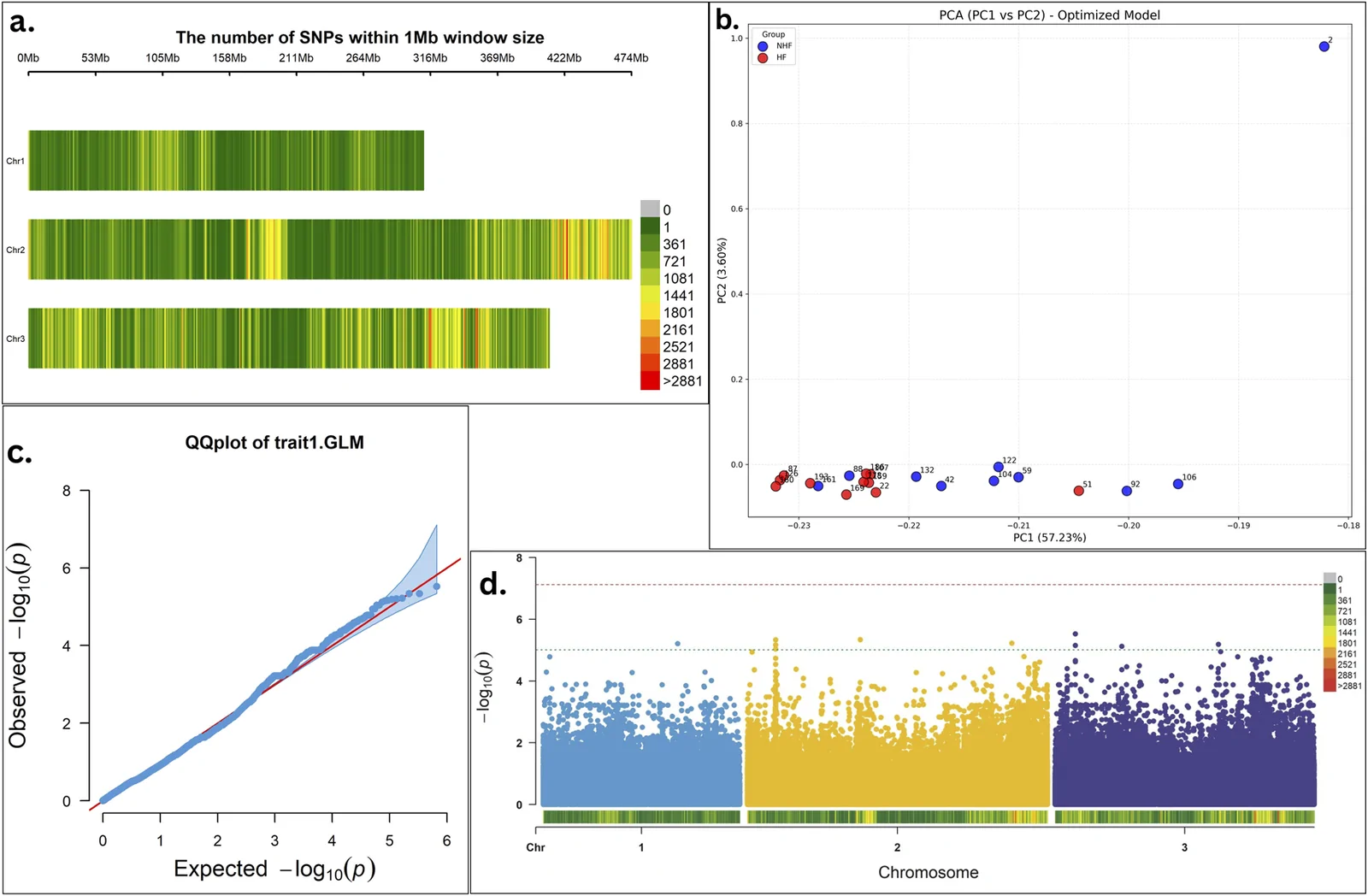
Genomic characterization of realized host-feeding behavior in field-collected *Ae. aegypti*. **(a)** Genome-wide SNP density heatmap across the three chromosomes of *Ae. aegypti* **(b)** Principal Component Analysis (PCA) of whole-genome SNP data from 21 *Ae. aegypti* individuals. **(c)** Quantile-Quantile (Q–Q) plot of observed versus expected −log_10_(p) values from the GWAS. **(d)** Manhattan plot displaying genome-wide association results across Chr1, Chr2, and Chr3. The color bar at the bottom of the Manhattan plot mirrors the SNP density scale from panel **(a)**. The red dotted line indicates genome-wide significance threshold and the blue dotted line indicates suggestive significance threshold.

Following joint genotyping and multi-step quality filtering (biallelic SNPs only; mean depth ≥10; MAF ≥0.05; HWE p ≥ 10^–3^; QUAL ≥30), a total of 661,519 high-quality SNPs were retained for downstream analysis. These were distributed across all three *Ae. aegypti* chromosomes, with SNP counts and densities as follows: Chr1 contained 115,994 SNPs at a density of 373.1 SNPs/Mb; Chr2 contained 270,005 SNPs at 569.1 SNPs/Mb; and Chr3 contained 275,520 SNPs at 672.3 SNPs/Mb. The overall mean SNP density was approximately 553.5 SNPs/Mb (Figure 1a). Chromosomes 2 and 3 showed elevated SNP density relative to chromosome 1.

### 3.3 Population structure analysis

Principal Component Analysis (PCA) was performed on an LD-pruned subset of the filtered SNP dataset to characterize genetic structure among the 21 *Ae. aegypti* samples and to identify stratification requiring correction in the GWAS model (Figure 1b). PC1 explained 57.23% of the total genetic variance, and PC2 explained an additional 3.60%. These two PCs together captured approximately 60.83% of the total variance, indicating a genomic dataset dominated by a single primary axis of genetic differentiation.

In the PC1 vs. PC2 biplot, NHF (non-human-blood-fed) and HF (human-blood-fed) samples clustered together along the primary axis, suggesting that phenotypic group membership does not correspond to the dominant axis of genetic divergence. However, notable outliers were observed, most prominently sample 2, which was strongly displaced along the positive tail of PC1. To appropriately adjust for this structure without over-parameterizing the model, and given the small sample size, a Scree plot was evaluated (Supplementary Figure S2). The variance explained dropped sharply after PC1 and effectively flattened out by PC3. We empirically tested models incorporating three, five, and 10 PCs to balance residual degrees of freedom with the control of population stratification. While the three-PC model exhibited remaining inflation (λ = 1.26), the inclusion of the first five PCs successfully reduced the genomic inflation factor to an optimal λ = 1.05. This five-PC model was selected as the optimal covariate threshold, as it adequately controlled for residual confounding while preserving 15 crucial degrees of freedom.

### 3.4 Genome-wide association analysis

Genome-wide association testing was performed using the GLM model incorporating the optimized selection of the first five PCs as fixed-effect covariates. The Q-Q plot (Figure 1c) showed that when–log_10_(*p*) value was in the range of 0–3, the observed data points closely followed the expected diagonal, and when–log_10_(*p*) exceeded 3, the observed points deviated slightly upward from the diagonal suggesting successful control for potential confounding factors such as population stratification. Consistent with the Q-Q plot, the genomic inflation factor (λ = 1.05) indicated minimal residual inflation and adequate correction for population structure. The Bonferroni-corrected genome-wide significance threshold was set at p < 7.56 × 10^−8^ (i.e., 0.05/661,519 SNPs). A suggestive threshold of p < 1.0 × 10^−5^ was additionally applied to capture candidate loci for functional interpretation, given the limited statistical power inherent to a sample of n = 21.

Under this 5PC model, no variants reached the stringent genome-wide significance threshold. However, the Manhattan plot (Figure 1d) identified 11 SNPs that crossed the suggestive significance threshold (p < 1.0 × 10^−5^) (Supplementary Table 1). These suggestive candidate loci were distributed across all three chromosomes. Notably, four suggestive SNPs on Chromosome 2 were located within or adjacent to LOC5577073, which encodes the actin-binding protein Supervillin (*Svil*). On Chromosome 3, two associated SNPs mapped to LOC5578948, encoding a disheveled-associated activator of morphogenesis-like protein (*DAAM*), and another highly suggestive variant was located in LOC23687755, which encodes G-protein coupled receptor 39. The functional relevance of these loci to host preference is not immediately apparent. These findings indicate that while population structure was successfully managed, the genetic architecture of the realized host-feeding preference (HF vs. NHF) cannot be definitively resolved within this specific cohort, underscoring the requirement for larger sample sizes to generate the statistical power necessary for detecting true biological associations.

### 3.5 Chromosomal inversions and host-feeding-associated gene content

**FIGURE 2:**
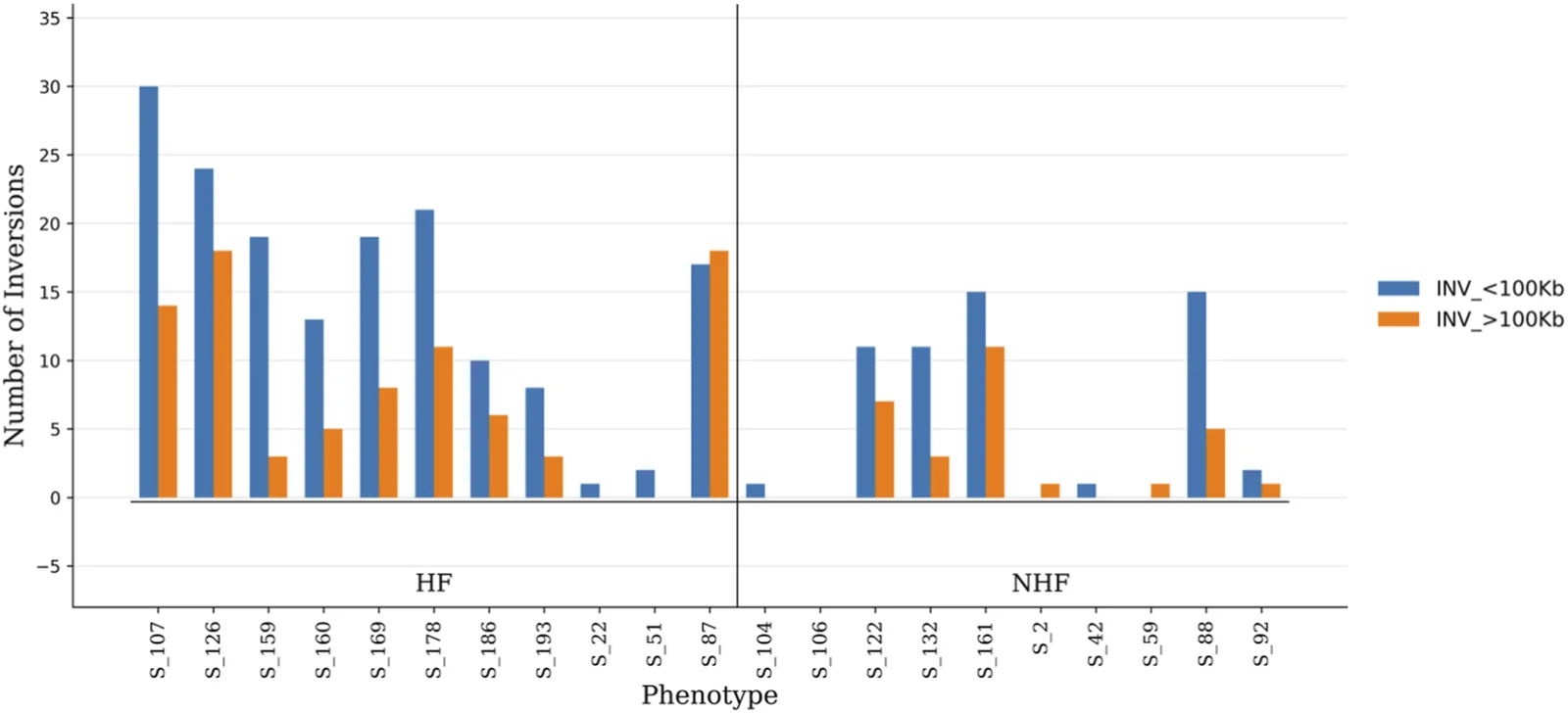
Per-individual chromosomal inversion counts across human-fed (HF) and non-human-fed (NHF) *Ae. aegypti* phenotypic groups.

Structural variant analysis using Delly v2.0 identified both small (<100 kb) and large (>100 kb) chromosomal inversions across the 21 samples (Figure 2). The HF group harbored a higher total number of inversion events compared to the NF group. Large chromosomal inversions are recognized as important drivers of adaptive divergence in insects, as they suppress recombination and can preserve co-adapted gene complexes under selection (Kirkpatrick and Barton, 2006). It was also evident from Timema stick insects that multi-megabase inversions are associated with adaptive color pattern differences that facilitate local adaptation to distinct host plants by maintaining allelic combinations resistant to recombination (Villoutreix et al., 2023).

To identify genes within inversion regions potentially relevant to host-feeding behavior, coding sequences (CDS) within all detected inversions were annotated and compared between the HF and NHF groups. Genes present in more than 60% of samples within a group were retained for further analysis. This analysis revealed that all genes found within inversions of the NHF group were also present within inversions of the HF group; however, a distinct and exclusive set of genes was uniquely captured within inversions of the HF group on chromosome 2 (Table 1). This asymmetry suggests that chromosome 2 inversions in human-blood-fed individuals may harbor gene combinations relevant to field realized host preference.

**TABLE 1:** List of genes found within large inversions on chromosome 2 of blood-fed *Ae. aegypti*.

GO terms	Gene name	Gene ID
Sensory organ development (GO:0007423)	Basic helix-loop-helix neural transcription factor TAP(*tap*)	5572211
Odorant binding (GO:0005549)	General odorant-binding protein 68(LOC5567749)	5567749
	Odorant receptor 31(*Or31*)	5577473
	Odorant receptor 33(*Or33*)	23687782
	Odorant receptor 6(Or6)	23687968
7TM_chemoreceptor superfamily (IPR013604)	Gustatory receptor 15(Gr15)	23687724
	Gustatory receptor 16(Gr16)	23687650
	Gustatory receptor 17(Gr17)	23687517
	Gustatory receptor 18(Gr18)	23687747
	Gustatory receptor 19a(Gr19a)	5574306
	Gustatory receptor 35(Gr35)	23687835
	Gustatory receptor 36(Gr36)	23687589
	Gustatory receptor 66(Gr66)	23687814

The HF-specific inversion region on chromosome 2 contained 13 genes assignable to three functionally coherent GO categories. First, *tap* (basic helix-loop-helix neural transcription factor; GO:0007423, sensory organ development) was present in 9 of 11 HF samples, consistent with a role in specifying or maintaining olfactory sensory neuron identity. Second, three odorant receptor genes, *Or6*, *Or31*, and *Or33*, along with *general odorant-binding protein 68* (*Gp68*) were captured within the inversion (GO:0005549, odorant binding). Odorant receptors in *Ae. aegypti* are the primary mediators of fine-grained host discrimination: females rely heavily on neurons expressing OR-family receptors for discrimination among host species, and females with mutations in the OR co-receptor *orco* fail to discriminate strongly between humans and animals (Zhao et al., 2022). The co-localization of multiple OR genes within a single HF-specific inversion is therefore a biologically compelling finding, as inversions suppress recombination and could maintain combinations of OR alleles that collectively enhance human-host detection. Third, a cluster of eight gustatory receptor genes, *Gr15*, *Gr16*, *Gr17*, *Gr18*, *Gr19a*, *Gr35*, *Gr36*, and *Gr66*, was found within the HF-specific inversion (IPR013604, 7TM chemoreceptor superfamily). Gustatory receptors in *Ae. aegypti* are not restricted to contact chemosensation; the CO_2_ receptor in *Ae. aegypti* is a heteromeric gustatory receptor complex comprising Gr1 and Gr3 subunits, and Gr3 mutants fail to detect CO_2_ or show CO_2_-evoked responses to heat and lactic acid, placing Gr family members as central mediators of long-range host localization (McMeniman et al., 2014). The discovery of eight additional Gr family members exclusively within HF inversions points to structural genomic variation as a potentially important mechanism linking chemoreceptor gene combinations to host preference phenotype. Taken together, the concentration of sensory organ developmental regulators, odorant receptors, OBPs, and gustatory receptors within a single HF-specific chromosomal inversion on chromosome 2 provides strong preliminary evidence for a genomic architecture in which co-adapted chemosensory gene combinations are maintained by suppressed recombination in human-preferring individuals.

## 4 Discussion

This study presents one of the first integrative genomic investigations of host-feeding behavior in field-collected *Ae. aegypti*, combining genome-wide association analysis (GWAS) with structural variant detection. Understanding the genetic determinants of human blood-preference is essential, as anthropophily is a primary driver of vectorial capacity for dengue, Zika, and chikungunya viruses. Our findings indicates that host preference could potentially be shaped by both polygenic SNP-level variation and a major chromosomal inversion harboring a functionally coherent chemosensory gene cluster, suggesting a possible supergene-like architecture underlying this behavioral trait.

### 4.1 Population structure and genomic background

Principal component analysis (PCA) revealed a strong primary axis of genetic variance among the sampled individuals, with PC1 and PC2 collectively explaining 60.83% of the total genomic variance (PC1 = 57.23%, PC2 = 3.60%). The PCA biplot demonstrates that the vast majority of samples form a single, continuous cluster distributed along PC1. Notably, non-blood-fed (NHF) and blood-fed (HF) individuals are completely intermixed within this main cluster, demonstrating that phenotypic group membership does not correspond to this dominant genetic structure. Furthermore, the variation along the secondary axis (PC2) is almost entirely driven by a single extreme outlier, NHF sample 2, which is markedly displaced from the rest of the cohort. This structuring likely reflects intraspecific variation within the single *Ae. aegypti* lineage present in India, where the ancestral African subspecies *Ae. aegypti* formosus has no recorded occurrence. Notably, Kumar et al. (2022) described a morphological variant, *Ae. aegypti* var. luciensis, co-existing with the type form across multiple regions of Tamil Nadu, India; despite differences in hind-tarsal scaling patterns, both forms were genetically indistinguishable by COI and ITS2 markers, confirming their conspecific status. The observed PCA scatter—specifically the continuous gradient along PC1 and the extreme isolation of sample 2 on PC2—may indicate fine-scale intraspecific genomic differentiation between such co-occurring morphological forms, high individual heterozygosity, or isolation-by-distance structuring previously documented across geographically distributed Indian *Ae. aegypti* populations (Sumitha et al., 2023; Sharma et al., 2026a; Sharma et al., 2026b), rather than ancestry-level subspecific divergence. Given the coexistence of these forms and their uncertain behavioral equivalence, detailed comparative studies on their bionomics, ecology, host-seeking behavior, and plausible differential roles in arboviral transmission are warranted.

### 4.2 GWAS candidate loci

The genome-wide association study identified several suggestive loci associated with field realized host-feeding behavior (HF vs. NHF) after applying population structure corrections. Optimizing the GLM to include only the first five PCs successfully controlled genomic inflation (λ = 1.05) while preserving mathematical stability.

While this adjustment eliminated false-positive artifacts, it revealed 11 suggestive candidate loci with observed suggestive significance at < 10^–6^ level (Supplementary Table 1) associated with the human feeding phenotype. Although these loci did not reach strict genome-wide significance (p < 10^–8^), an expected limitation given the constrained statistical power of the small cohort, the identified candidate genes may provide some biologically plausible targets for behavioral regulation.

Most notably, a suggestive SNP on Chromosome 3 was mapped to a G-protein coupled receptor (GPCR 39). GPCRs are critical components of insect neurobiology, playing central roles in regulating physiological pathways, neurodevelopment, reproduction, behavior and metabolism (Hii et al., 2002; Li et al., 2014). Although G protein-coupled receptors (GPCRs) constitute the major class of olfactory receptors (ORs) in mammals mammalian (Mombaerts, 2004), *Drosophila* ORs exhibit no obvious sequence homology to GPCRs, despite possessing a predicted seven-transmembrane domain architecture. Nevertheless, the established involvement of GPCR-type olfactory receptors in odorant detection across both vertebrates and invertebrates provided a strong basis for investigating their potential role in host-seeking behavior (Spehr and Munger, 2009). Additionally, other suggestive SNPs were mapped to genes encoding actin-modulating proteins, including Supervillin (*Svil*) and a DAAM-like protein. While their direct role in mosquito feeding requires further validation, cytoskeletal dynamics are essential for sensory neuron function and synaptic plasticity.

These results underscore the critical need for larger, adequately powered cohorts to elevate these suggestive neurobehavioral signals to genome-wide significance and fully disentangle the genetic drivers underlying mosquito feeding preferences.

### 4.3 Chromosomal inversion and possible supergene architecture

The key finding of this study is the identification of an HF-exclusive chromosomal inversion on chromosome 2 containing 13 genes spanning three chemosensory GO categories: sensory organ development (tap), odorant binding (Or6, Or31, Or33, Gp68), and 7TM chemoreception (Gr15–Gr19a, Gr35, Gr36, Gr66). Chromosomal inversions suppress recombination between alternative arrangements, enabling co-adapted allele combinations to persist as intact supergene-like units (Kirkpatrick and Barton, 2006). In insects, chromosomal inversions create reverse-order genomic regions that are protected from meiotic recombination, and large clusters of genes within inversions are inherited almost intact as a single unit, facilitating rapid divergence of allelic sets between alternative arrangements.

In *Anopheles gambiae*, the 2La inversion is the canonical precedent, it has been repeatedly associated with ecological adaptation and host preference, with inversion frequency correlating with human-biting rates in field populations (Ayala et al., 2014; Riehle et al., 2017). The most common mechanism by which inversions operate appears to be divergence of allelic sets between alternative arrangements due to suppression of recombination between arrangements. However, the available data on the precise mechanisms of the functional effects of inversions are still limited, and it is too early to make conclusive generalizations.

The odorant receptors found within this inversion, *Or6, Or31*, and *Or33*, are functionally compelling candidates. Foundational work by McBride et al. (McBride et al., 2014), established that upregulation and allelic variation of AaegOr4 drives increased sensitivity to sulcatone, a human-specific volatile, as the primary molecular basis of anthropophily in domestic *Ae. aegypti*. While Or4 was not independently highlighted in our GWAS, the strong association of multiple OR loci within a single non-recombining inversion indicates that host preference is likely a polygenic olfactory trait mediated by a synergistic repertoire of receptors tuned to human volatiles, rather than a single-gene effect. Further, the presence of *tap*, a bHLH transcription factor expressed exclusively in chemosensory neurons during differentiation and most closely related to vertebrate neurogenin and neuroD (Ledent et al., 1998), governs the development of both adult antennal olfactory organs and gustatory bristles, with its ectopic expression directly altering behavioral responses to tastants (Gautier et al., 1997). Its co-inheritance alongside *Or6*, *Or31*, *Or33*, odorant-binding protein Gp68, and gustatory receptors (Gr15–Gr19a, Gr35, Gr36, Gr66) within a single non-recombining chromosomal unit suggests that co-adapted variation in chemosensory neuron specification and receptor repertoire is preserved simultaneously, a particularly efficient genomic mechanism for entrenching coordinated anthropophilic host-seeking. The gustatory receptor cluster further extends the chemosensory scope of this region; CO_2_ receptor subunits Gr1 and Gr3 are already established as critical mediators of long-range host localization in *Ae. aegypti* (Sanford et al., 2013; Sparks et al., 2013) supporting the functional plausibility of the broader Gr family members identified here.

**FIGURE 3:**
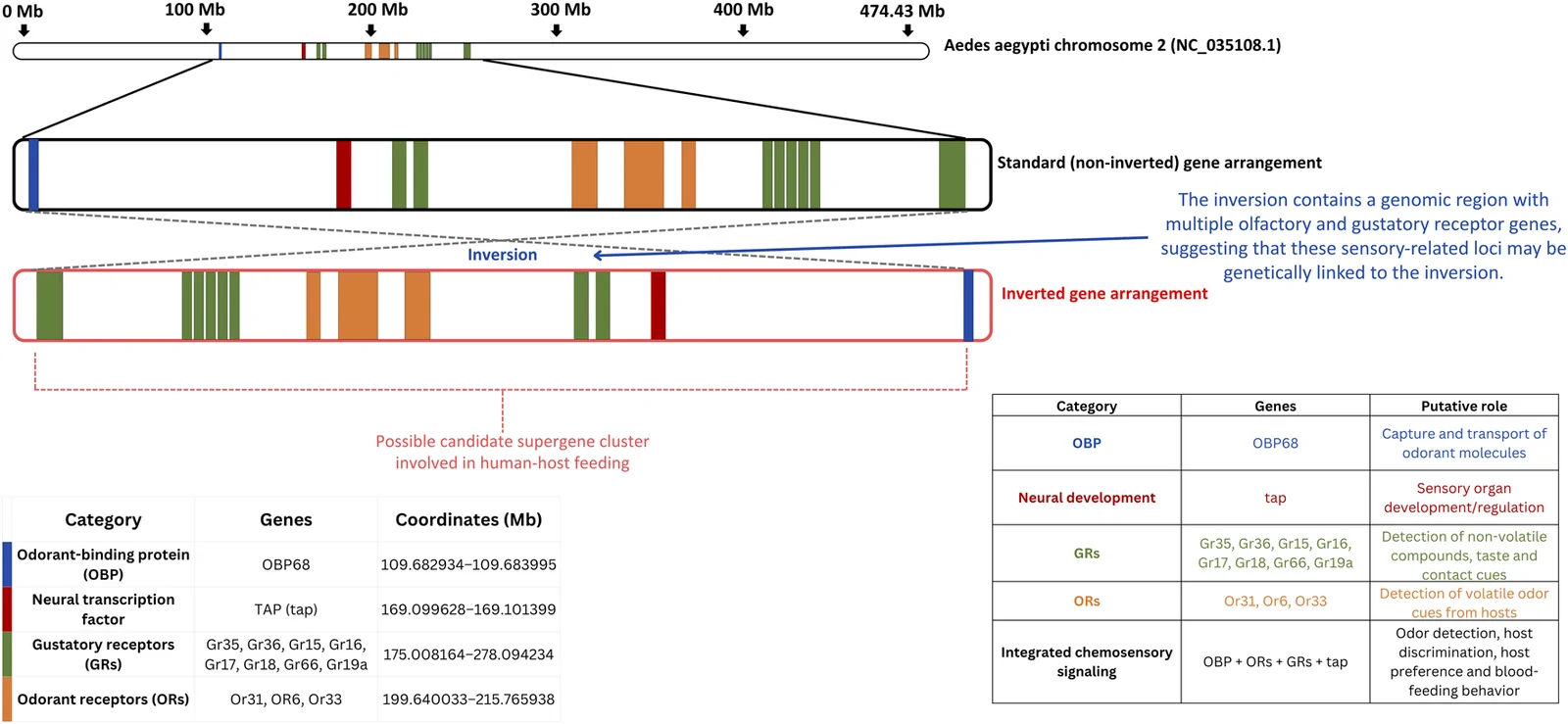
Conceptual framework of a potential supergene cluster on *Aedes aegypti* chromosome 2 (NC_035108.1). This proposed model compares standard and inverted gene arrangements, illustrating a chromosomal inversion encompassing a genomic region rich in sensory-related loci. The hypothesised mechanism suggests that the genetic linkage of odorant-binding proteins (OBP68), neural transcription factors (tap), gustatory receptors (GRs), and odorant receptors (ORs) within this inversion facilitates chemosensory signalling (OBP + ORs + GRs + tap).

An important alternative interpretation is that this inversion reflects a broader sensory adaptation rather than a mechanism specific to human-host preference. The genes captured within the inversion, odorant receptors, gustatory receptors, an odorant-binding protein, and the neural specification factor *tap*, collectively regulate a wide range of chemosensory-driven behaviors in mosquitoes, including sugar and nectar foraging, mate location, oviposition-site selection, and CO_2_-mediated host localization, in addition to fine-scale host discrimination. The concentration of chemosensory genes within a single non-recombining block does not, on its own, establish that this structural variant evolved specifically for anthropophily; it may instead represent a co-adapted chemosensory module affecting multiple ecologically relevant behaviors, of which host discrimination is one. Distinguishing between these hypotheses will require functional and behavioral assays targeting each candidate gene independently. Here, we propose a Conceptual framework for a potential supergene cluster on *Aedes aegypti* chromosome 2 (NC_035108.1) that drives human-host feeding behaviour. This proposed model contrasts standard and inverted gene arrangements (Figure 3). Chromosomal inversions suppress homologous recombination within the inverted region, thereby reducing the genetic reshuffling of linked loci. When the inversion encompasses multiple genes involved in olfactory and gustatory perception, reduced recombination can preserve specific combinations of alleles across these loci. Consequently, these co-adapted genetic variants may be inherited as a relatively stable haplotypic block, facilitating the maintenance and transmission of advantageous sensory phenotypes across generations (Sharakhov and Sharakhova, 2024).

### 4.4 Limitations and strength

Several limitations must be acknowledged. The sample size of n = 21 is the principal constraint; GWAS is inherently underpowered at this scale, and reported effect sizes are subject to upward bias (“winner’s curse”) (Palmer and Pe’er, 2017), necessitating independent replication in larger field cohorts. As inversion detection relied exclusively on short-read paired-end sequencing evidence and a single calling algorithm (Delly), the reported chromosome 2 inversion should be regarded as a candidate structural variant. Cross validation with an independent caller and/or experimental breakpoint confirmation *via* long-read sequencing (PacBio HiFi/Oxford Nanopore) or Hi-C chromatin contact mapping would substantially increase confidence in this candidate inversion. We have not directly demonstrated suppressed recombination within the candidate inversion (e.g., *via* comparative LD decay analysis between inversion arrangements or fine-scale recombination-rate mapping). Therefore, the term ‘supergene’ used to denote a candidate/putative architecture, consistent with the presence of multiple functionally related genes in a structurally distinct region. Finally, all candidate loci, including inversion-associated *OR*, *Gr*, and *tap* genes, require functional validation. This study did not include behavioral assays (e.g., dual-choice olfactometer or Y-tube assays) to directly measure innate host preference under controlled conditions. The genetic associations reported here are therefore inferred from field blood-meal identification alone and represent associations with realized host-feeding outcome, not direct evidence of an innate preference allele. Controlled behavioral validation of candidate loci (e.g., in colonized lines segregating for the chromosome 2 inversion) is an essential next step to confirm a causal link to host preference.

Despite these constraints, the study offers substantive contributions to the field. To our knowledge, this is the first investigation to integrate GWAS and structural variant analysis to investigate host-feeding behavior in field-collected *Ae. aegypti*. The convergence of SNP-level associations in chemosensory gene regions with an HF-exclusive chromosomally inverted supergene-like block substantially strengthens biological plausibility despite the modest sample size. While anthropophily determines vectorial capacity of mosquito, and the identified chromosome 2 inversions, which can potentially be genotypable *via* simple diagnostic markers, could serve as a field-deployable proxy for human-biting propensity in surveillance programs. The *OR* and *Gr* loci within the HF inversion further represent priority molecular targets for next-generation repellent and lure-based vector control strategies.

## 5 Conclusion

This study provides the first genome-wide characterization integrating SNP associations and chromosomal inversion polymorphisms with its potential role in the host-feeding behavior among field-collected *Ae. aegypti*. GWAS identified four suggestive SNPs on Chromosome 2 were located within or adjacent to LOC5577073, which encodes the actin-binding protein Supervillin (*Svil*). On Chromosome 3, two associated SNPs mapped to LOC5578948, encoding a disheveled-associated activator of morphogenesis-like protein (*DAAM*), and another highly suggestive variant was located in LOC23687755, which encodes G-protein coupled receptor 39. Most critically, a potential HF-specific chromosomal inversion on chromosome 2 containing a co-inherited cluster of odorant receptors, gustatory receptors, odorant-binding proteins, and a sensory developmental regulator consistent with a supergene model of host preference analogous to the *An. gambiae* 2La inversion. This architecture suggests that suppressed recombination preserves co-adapted chemosensory allele combinations in human-blood-feeding mosquitoes, representing a plausible evolutionary mechanism for the entrenchment of anthropophily. A logical next step is differential gene expression profiling (e.g., RNA-seq of antennal/olfactory tissue) comparing HF and NHF individuals, focused on the chemosensory genes identified within the chromosome 2 inversion (*tap*, *Or6*, *Or31*, *Or33*, *Gp68*, and the *Gr15–Gr19a/Gr35/Gr36/Gr66* cluster). Such expression data, combined with behavioral choice assays, would substantially strengthen the causal link between this candidate genomic architecture and host-feeding phenotype. These findings could establish a genomic foundation for molecularly informed surveillance. These inversion based markers could offer potential proxies for population-level human-biting tendency, and candidate *OR* and *Gr* loci serving as targets for next-generation repellent and vector control strategies.

## Data Availability

The whole-genome sequencing datasets generated during this study have been deposited in the NCBI Sequence Read Archive (SRA) under BioProject accession number [PRJNA1479202].
