# Supplementary material for: Genomic analysis identifies candidate supergene architecture for host-feeding behavior in wild Aedes aegypti dengue vector mosquitoes
Source: Front Genet. 2026 Sep 9;17:1910877. doi: 10.3389/fgene.2026.1910877 (PMC13597056; doi:10.3389/fgene.2026.1910877)
Supplement: Supplementary file 1 [file DataSheet1.docx]

**Supplementary Files**

**Supplementary Figures**

**Figure S1: IGV visualization of a candidate inversion breakpoint.**

The upper track shows alignment from the non-inversion sample, whereas the lower track shows alignment from the inversion sample at approximately CM008044.1:45,248,351–45,248,526. Compared with the non-inversion sample, the inversion sample displays an increased concentration of split and/or discordantly oriented reads near the candidate breakpoint.

**Figure S2:** **Scree plot of principal component analysis (PCA) on linkage disequilibrium-pruned genome-wide SNP data from 21 field-collected *Ae. aegypti* individuals.**

**Supplementary Table:**

**Supplementary Table 1: Suggestive loci (**$P<1.0\times{10}^{-5}$**) associated with field realized host-feeding behavior (HF vs. NHF) in *Ae. aegypti*.**

| **Chromosome** | **SNP Position** | **Reference Allele** | **Alternate Allele** | **Effect** | **SE** | **-log_10_(p)** | **Gene** | **Gene Name** |
| --- | --- | --- | --- | --- | --- | --- | --- | --- |
| 1 | 212537996 | C | G | -0.77 | 0.11 | 6.23E-06 | RNA-seq intron-spanning reads |  |
| 2 | 44689669 | A | G | 0.72 | 0.10 | 6.80E-06 | LOC5577073 | Supervillin (*Svil*) |
| 2 | 44762032 | C | T | 0.73 | 0.10 | 4.66E-06 | LOC5577073 |  |
| 2 | 44772282 | A | C | 0.70 | 0.10 | 9.21E-06 | LOC5577073 |  |
| 2 | 44772292 | T | A | 0.70 | 0.10 | 9.21E-06 | LOC5577073 |  |
| 2 | 178539341 | G | A | -0.48 | 0.07 | 4.62E-06 | RNA-seq intron-spanning reads |  |
| 2 | 417902065 | A | G | -0.72 | 0.10 | 6.04E-06 | RNA-seq intron-spanning reads |  |
| 3 | 32086971 | G | A | -0.56 | 0.08 | 7.10E-06 | LOC5578948 | Disheveled-associated activator of morphogenesis-like protein (*DAAM*) |
| 3 | 32128912 | A | G | -0.53 | 0.07 | 2.99E-06 |  |  |
